# Building integrated diffusers’ area ratio optimization

**DOI:** 10.1038/s41598-024-55091-9

**Published:** 2024-02-24

**Authors:** Abdel Rahman Elbakheit

**Affiliations:** Department of Architecture and Building Science, King Saud University, Riyadh, Saudi Arabia

**Keywords:** Diffuser area ratio, Wind energy augmentation, Building integrated diffusers, Diffuser sizing, Wind energy optimization in buildings, Diffuser optimization, Energy science and technology, Engineering

## Abstract

This paper presents an investigation into the effect of area ratio parameter of diffusers on its energy output through power coefficient C_p_. This parameter has effect both on diffusers’ energy yield, besides diffuser’s size for architectural integration prospects. A systematic increase in diffusers area ratio is adopted following standardized diffuser profile presented by NACA 1244 aerofoil. A series of area ratios were investigated (i.e., 1.25, 1.5, 1.75, 2, 2.5, 3 and 3.5). Area ratio of 1.5 (i.e., outlet/inlet, 0.75 m/0.50 m) exhibited the highest power coefficient C_p_ of 4.2, in addition to achieving highest resulting velocity of 25.8 m/s under incident velocity of 16m/s. Considerable wind separation inside inner walls of diffusers occurred from area ratio 1.75 onwards, which impacted resulting velocities. Simulations performed with ANSYS CFD Academic to standalone diffusers. A series of incident velocities employed from 1 to 16 m/s that resulted in velocity increase by 120–156% respectively.

## Introduction

**Figure 1:**
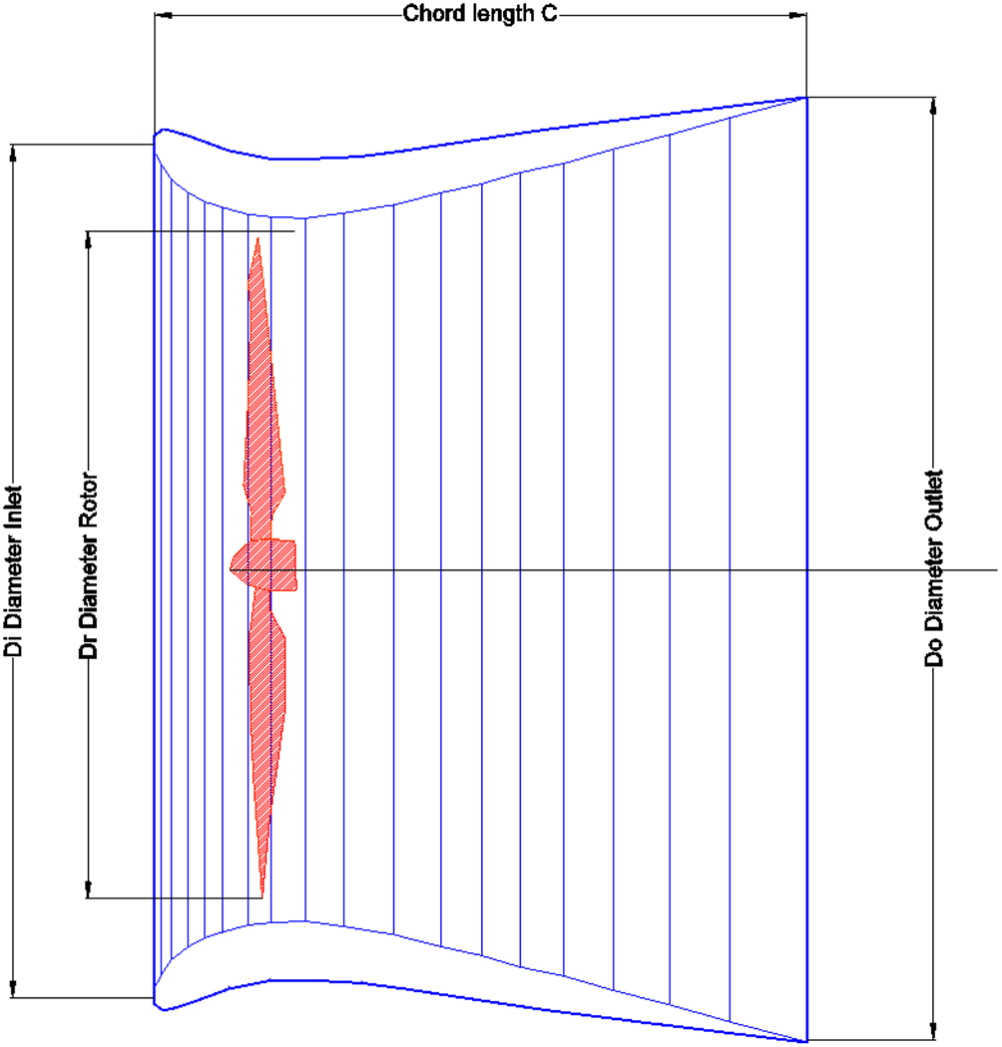
Igra’s diffuser profile^13^.

Wind energy is one of the very cleanest and readily available energy resources within the built environment^1^. With high growth of cities globally^2^ that necessitate increase in energy demand and ensued CO_2_ emissions; Building integrated wind turbines provides opportunities for providing valuable renewable energy within the built environment^3^. Thus, satisfying energy demand in a sustainable way and reducing CO_2_ emissions within the built environment. Furthermore, reducing power transmission losses^4^. The amalgamation of these efforts would hopefully reduce the production of CO_2_ and slow global warming and climate change^5^. However, many challenges are faced in this process^6^ due to the variability of wind patterns and the low magnitude of wind speeds posed by the presence of buildings and other obstacles. Diffuser’s, shroud’s and aerofoil’s^7,8^ wind augmented turbines provide an opportunity to solve these problems by accelerating wind flows to confined spots in a controlled manner. Thus, reducing the effect of low velocities and turbulences around buildings. Many researchers provided experimental and theoretical studies on these diffusers and shrouds their components^9^, types^10^, optimizations^11^ and underlying principles^12^. One of the key factors influencing performance of diffusers is the area ratio factor, which is diffuser outlet area over inlet area in Fig. 1. This factor also dictates the size of the diffuser and eventually it presence within the built environment. So optimizing this factor provides an insight on how efficient the diffuser is, as well as how big is its size is (3). Therefore, this paper may fill-in the gap present in identifying which diffuser to choose? Based on its size or performance. (i.e., optimizing the diffuser for optimum performance with optimum size). In other words, providing highest energy yield from potentially the smallest possible diffusers sizes.

## Material and methods

### Iqra’s profile and parameters

In addition, Igra^14^ proposed some remedies to flow separation present in the lee side of the diffuser resulting from the increase in area ratio. Obviously, flow separation leads to reduction in power generation. This is another reason to investigate the likely region where this separation occurs. However, some researchers^15^ have shown that a small diffuser (i.e., having low area ratios), performance may be much enhanced above Betz limit by optimizing the rotor or turbine blades. A comparison between numerous researchers’ work involving different diffuser area ratios was also undertaken by Ref.^9^. However, there was not a systematic incremental increase in area ratios, neither that other parameters affecting performance kept neutral from the comparison. In this paper, these two issues dealt with systematically.

### Mathematical methodology

The parameters of diffusers under consideration in this study presented in Fig. 1. Namely, area ratio parameter that is area of diffuser’s outlet over area of diffuser’s inlet. Other parameters such as Chord length C and sectional profile kept intact as possible. The main idea is to investigate the area ratio parameter of a generic cross-sectional profile to see it’s effect on diffuser’s power generation prospects as well as it’s expected size.

So no cross-sectional profile optimizations^16^ are included in this study.

The second parameter on which the performance of the diffuser is gauged is the power co-efficient C_p_, which denote the ratio between the power generated by the diffuser to that is generated by a bare turbine determined as follows:

1 $$Cp=\frac{{\text{Power\, by\, diffuser}} }{0.5{U}^{3} {A}\rho }$$

where U is the mainstream air velocity m/s, A turbine swept area in m^2^ and ρ is air density in kg/m^3^.

### Model development

#### Model design

**Figure 2:**
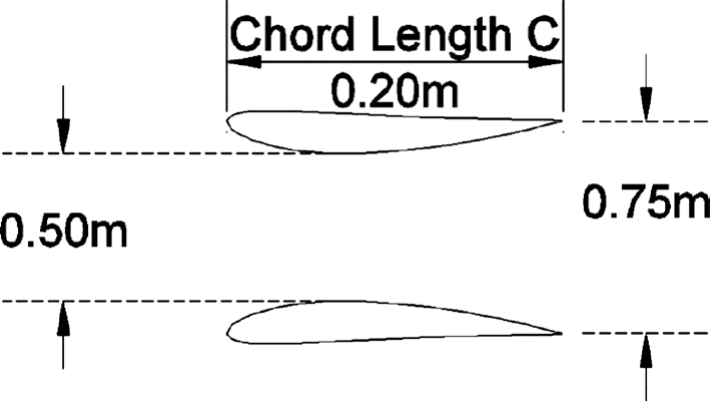
A schematic cross section of the 3D experimental model used for the numerical validation study adopted here as a 3D virtual model, which resembles model B without flap by Igra^14^.

Numerical simulation adopted employed the educational version of ANSYS workbench FLUENT to simulate effect of diffuser outlet/inlet ratio on expected power return. NACA 1244 aerofoil introduced to serve as diffuser’s cross sectional profile. The NACA 1244 profile used has a convex shape that accelerate air and provide lift force, therefore employed to all inner side of studied diffusers’ area ratios. However, this convex face is employed to outside in the diffuser with area ratio of 1.25 case only to keep the area ratio parameters intact. Other area ratios have the convex face of the aerofoil to the inner side of diffuser as Table 1 reveals. The simulation fixed the chord length C for all diffusers (i.e., distance C, in Figs. 1 and 2) to 1.639 m while, increasing the area ratio from 1.25 up to 3.5 as stipulated in Table 1.

**Table 1:** Area ratios of diffusers under study.

Area ratio	1.25	1.5	1.75	2	2.5	3	3.5
Inlet	0.3	0.5	1	1.5	2	1	1
Outlet	0.375	0.75	1.75	3	5	3	3.5
Side							
Sections							
3D

#### Meshing

**Figure 3:**
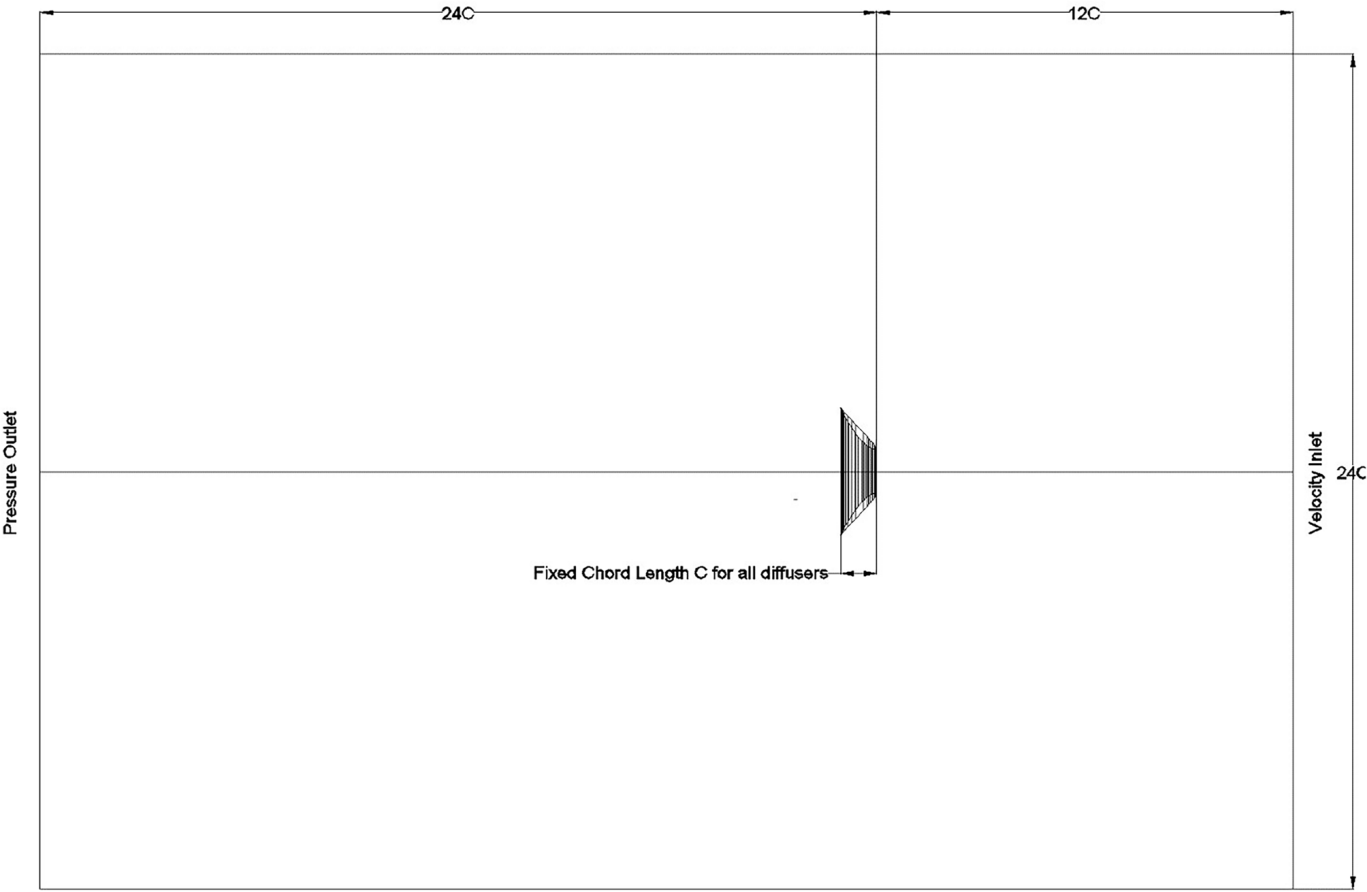
Domain size multiples of chord length C.

**Figure 4:**
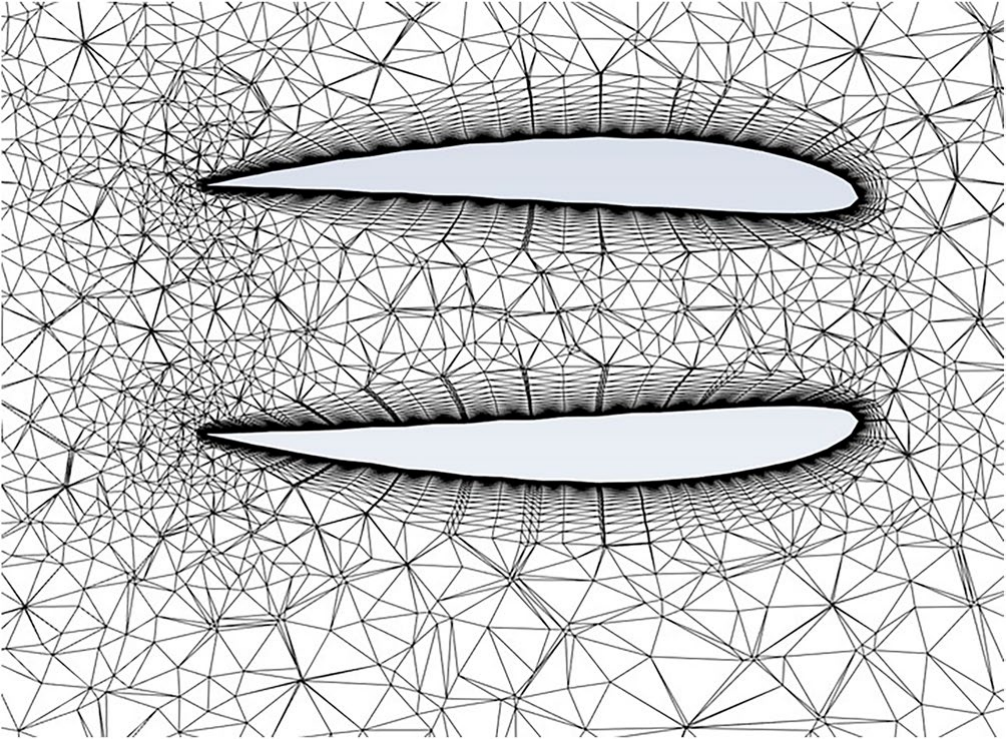
Inflation layers around the diffuser.

The grid meshing used a 3D tetrahedron mesh to the domain in Fig. 3, with a longitudinal section through the diffuser’s mesh presented in Fig. 4. This figure also reveals that the grid is extra refined around the diffuser with 20 inflation layers for further accurate description to the flow around the diffuser.

#### Boundary conditions applied

Boundary conditions involved velocity inlet for the range of studied velocities, Asymmetry condition to the sides of the domain and pressure Outlet. Model and other details of the simulation presented in the simulation methodology in point 2.5.

### Grid independence test

A further investigation to validity of the simulation domain undertaken by examining the number of cells within the domain. For this end, a 3D domain selected for its accuracy an efficiency. Three grid sizes tested against experimental work to assess the effect of grid size on obtained simulation results to stablish grid dependence. Fine grid of just in excess of 500,000 cells obtained the most accurate simulated results of 0.444 for thrust coefficient with a difference of 0.01 from experimental results, thus agrees well with experimental results as Table 2, reveals.

**Table 2:** Grid study for validation of 3D model in ANSYS FLUENT.

Grid	No. of cells	Force co-efficient simulated C_p_	Force co-efficient experiment C_p_
Coarse	153,544	0.392	0.434
Medium	242,885	0.415	0.434
Fine	502,011	0.444	0.434

### Simulation methodology

Simulation parameters employed in the investigation invloved a Pressure–velocity coupling second order, coupled scheme. Furthermore, steady state condition is activated under the standard turbelnece modle SST-K epsilon’. Most cases converged at about 100–1000 iterations.

## Results and discussion

### Grid convergence study

Determining the ordered discretization error of the grid gives confidence in the accuracy of the results and estimated error magnitude. To determine this, the Roaches^17^ Grid Convergence Index GCI^18^ can be calculated as follows:

From Table 2, Grids 3 and 2. Results of simulated force C_p_

2 $$\begin{aligned} {\text{The order }}\left( {\text{P}} \right) & = {\text{Ln }}\left[ {\left( {0.{444} - 0.{415}} \right)/\left( {0.{415} - 0.{392}} \right)} \right]/{\text{Ln2}} \\ & = {\text{Ln }}\left[ {0.0{29}/0.0{23}} \right]/{\text{ Ln2}} \\ & = 0.{334419}0 \\ \end{aligned}$$

3 $${\text{GDI}}12= 1.25 \left|(\frac{0.392-0.415)}{0.392}\right|/{(2}^{0.3344190}-1)100\%=0.281143707$$

4 $${\text{GDI}}23= 1.25 \left|(\frac{0.415-0.444)}{0.392}\right|/{(2}^{0.3344190}-1)100\mathrm{\%}=0.33483935$$

Now, we can check that the solution is within the asymptotic range of convergence.

$$={(0.33483935/2}^{0.3344190}x0.281143707=0.944578$$

0.944578, this figure indicates that the solution is within the asymptotic range of convergence; and that grid dependence is reached for the solution for grid size in the region of 500,000 cells or more. Source of error may be the maximum limit on cells number in the educational version of ANSYS FLUENT.

### Validation study

**Figure 5:**
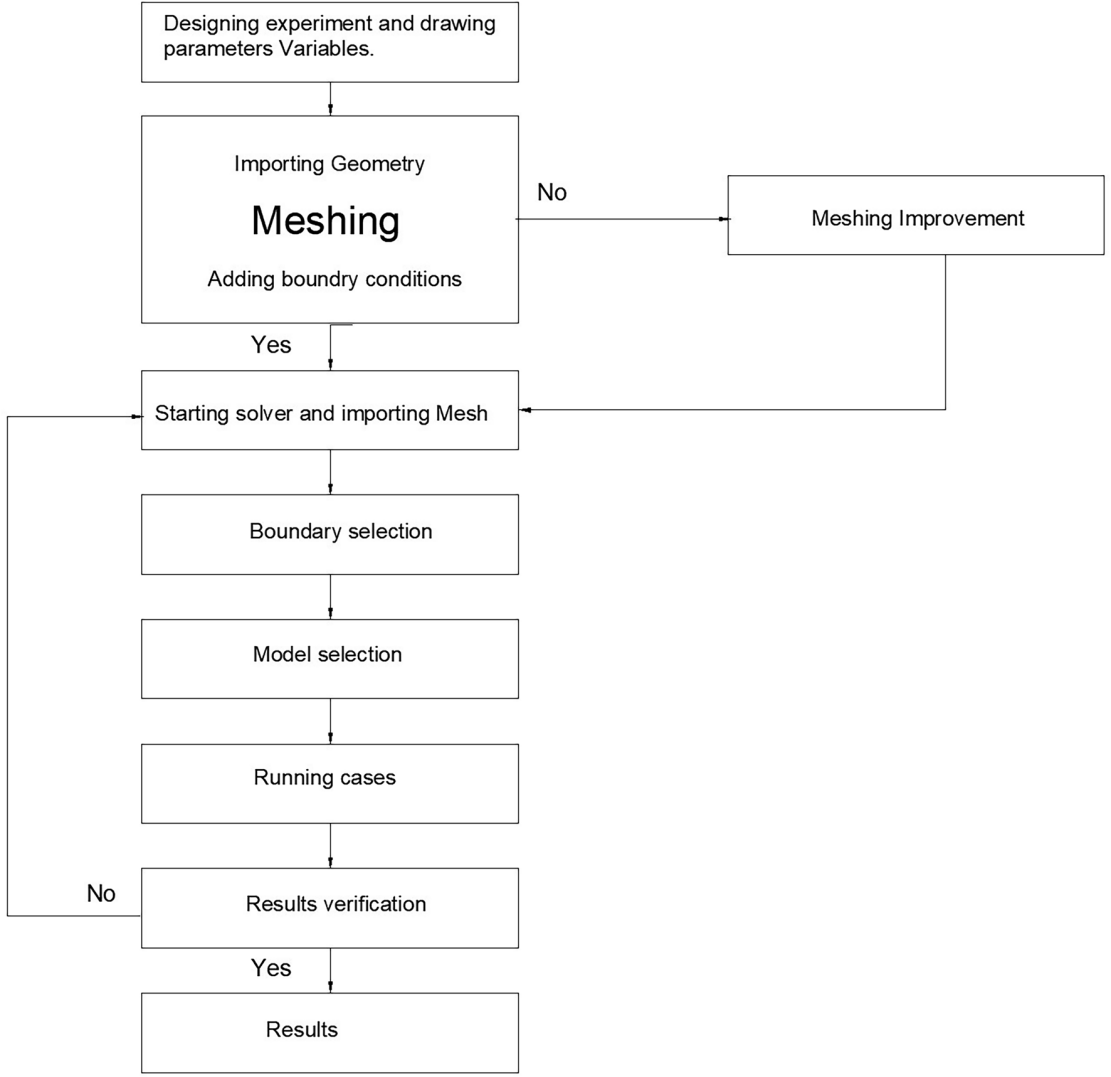
Flowchart describing the steps of the simulation.

In order to validate the simulation against previously reported data, Igra’s^19^ experimental model (B)’s results were chosen due to its similarity to the design adopted in this study without radial-gap, presented in Fig. 2. Simulation domain size presented in Fig. 3. The domain size is built to be of 12-times the chord length before the diffuser from front and sides and 24-times the cord in the leeside according to Ref.^13^ to naturalize domain dependence. The conditions of Igra’s experiment involved an inlet velocity of 32 m/s yielded thrust coefficient of 0.434. These conditions were applied to the constructed domain to compare the outcome of the simulation against the practical experiment as a validation point. A flowchart describing sequence of the steps of the simulation presented in Fig. 5.

### Variation of resulting velocities with incident velocities for various diffusers’ area ratio

**Figure 6:**
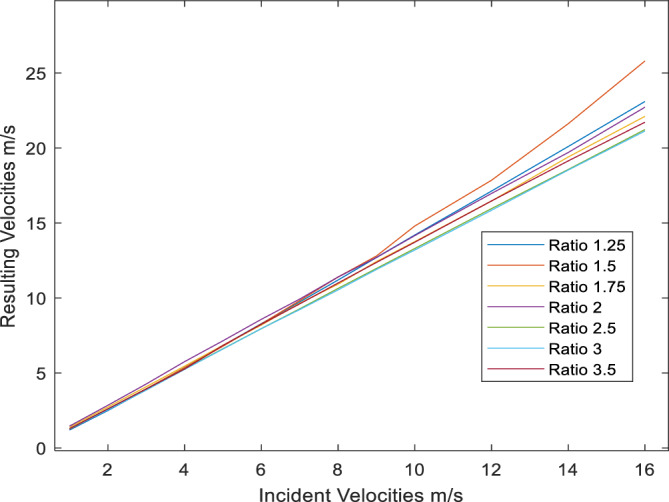
Resulting velocities with change in diffusers’ area ratio.

**Figure 7:**
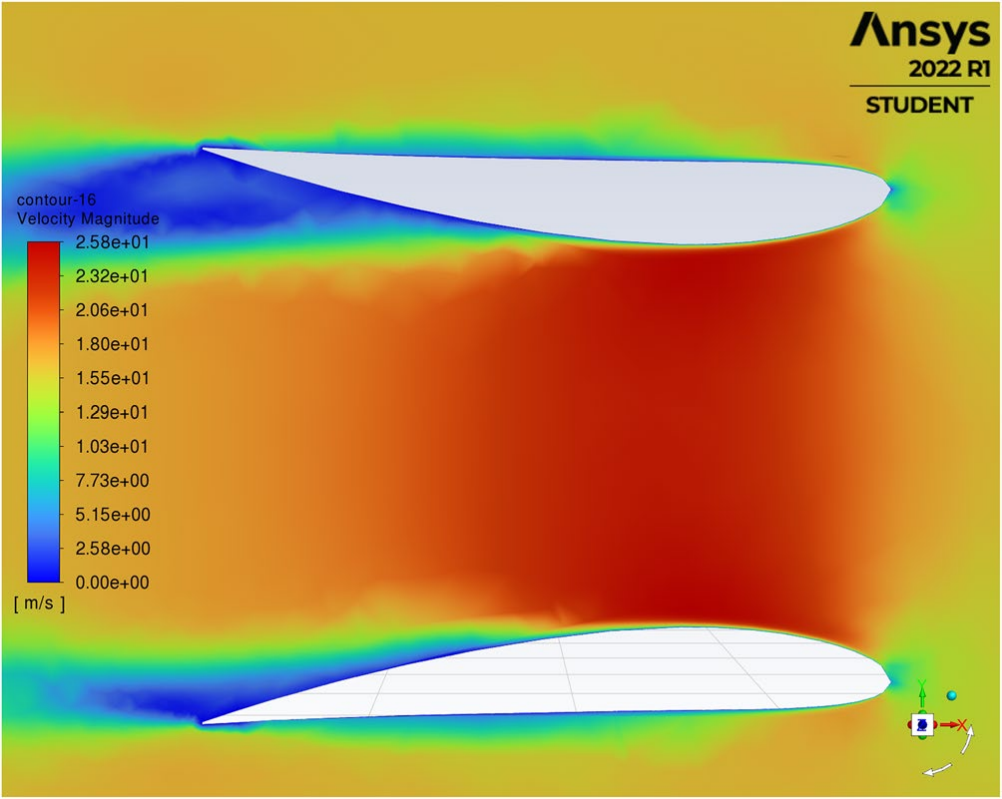
Velocity contours distribution for area ratio of 1.5 under incident velocity 16 m/s.

A comparison of the resulting velocities obtained from varying the area ratio of the diffusers shown in Table 1, after fixing the chord length to 1.639m and using NACA 1244 aerofoil as profile section for all diffusers. Figure 6 below depicts the effect of increasing the area ratio from 1.25 to 3.5. All area ratios experienced an increase in the resulting velocities with the increase in area ratio; however, area ratio of 1.5 produced the highest resulting velocities for the same incident velocities from 10 m/s and higher velocities. With the highest reported resulting velocity of 25.8 m/s under incident velocity of 16 m/s, Fig. 6. The velocity contours distribution of this highest recorded resulting velocity presented in Fig. 7, which reveals that all the shorter distance of the diffuser opening subjected to influence of the highest resulting velocity. However, the figure also reveals start of formation to flow separation at the trailing edge of the diffuser at the leeside. Fortunately, this separation had no effect on resulting velocity at this case. However, it would develop to be detrimental for resulting velocities for the succeeding cases as Table 3 reveals.

**Table 3:** Development of flow separation with increase of area ratio.

Area ratio	1.25	1.5	1.75	2	2.5	3	3.5
Resulting velocity m/s for 16 m/s	23.1	25.8	21.11	21.72	21.23	22.13	22.72
increase	144.38%	161.25%	138.19%	142%	132.69%	132.0625%	135.75%
Sections							
CFD							

### Variation of power coefficient (C_***p***_) with incident velocities for various diffusers’ area ratio

**Figure 8:**
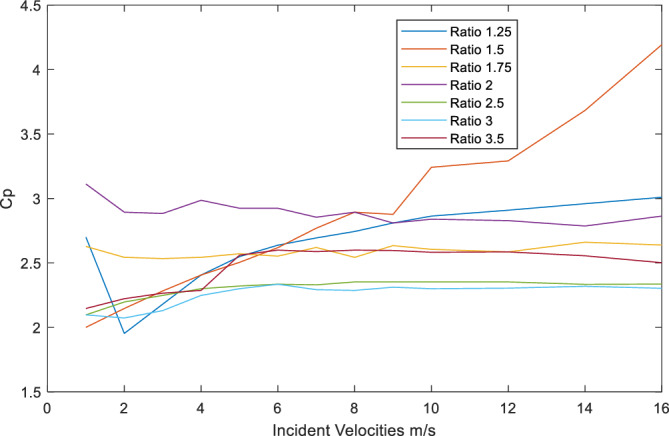
Power coefficient C_p_ under incident velocities.

The results of power coefficient C_p_ with the increase of incident velocities or mainstream velocities on diffusers presented in Fig. 8, below. The figure shows a steady and stable C_p_ for all tested diffuser’s area ratios that ranges from twice to three times the power of a bare turbine. With the increase of incident velocities from diffusers of area ratios 1.25, 1.75, 2, 2.5, 3 and 3.5 respectively. However, results of C_p_ for area ratio of 2 and 3 is slightly lower to about 1.7 time the power of a bare turbine at incident velocity of 2 m/s. While, area ratio of 1.5 maintained a gradual increase in C_p_ above the bare turbine to 4.2 times the power of a bare turbine at incident velocity of 16 m/s.

As a matter of a fact, this area ratio (i.e., 1.5) profile is the only profile that has a consistent increase of power at every given incident velocity up 10 m/s and beyond. Further increase of incident velocity from 10 m/s resulted in a near exponential increase in resulting C_p_ for this case.

### Development of flow separation with increase of area ratio

When consulting the whole picture of wind velocity profiles of all diffusers from Fig. 5 and Table 3. From Table 3 above we can observe that velocity contours of all cases under incident velocity of 10 m/s inlet velocity (i.e., at the bottom raw of the table), where the flow slightly separated in the leeside from area ratio of 1.5 onwards. At about area ration of 1.5 the maximum resulting velocity is obtained. However, with the increase in area ratio further than 1.5 resulting velocities started to decline, as it is affected by flow separation. This table (i.e. Table 3) also presents the resulting velocities for all cases under inlet velocity of 16 m/s, all cases obtained increase in resulting velocities with the maximum obtained for area ratio of 1.5 at 25.8m/s with an increase excesses 161%. As a validation to these results, Refs.^15,20^ reported best performance for their diffusers’ investigations at area ratio of 1.5.

### Effect of diffuser’s integration into building facades

**Figure 9:**
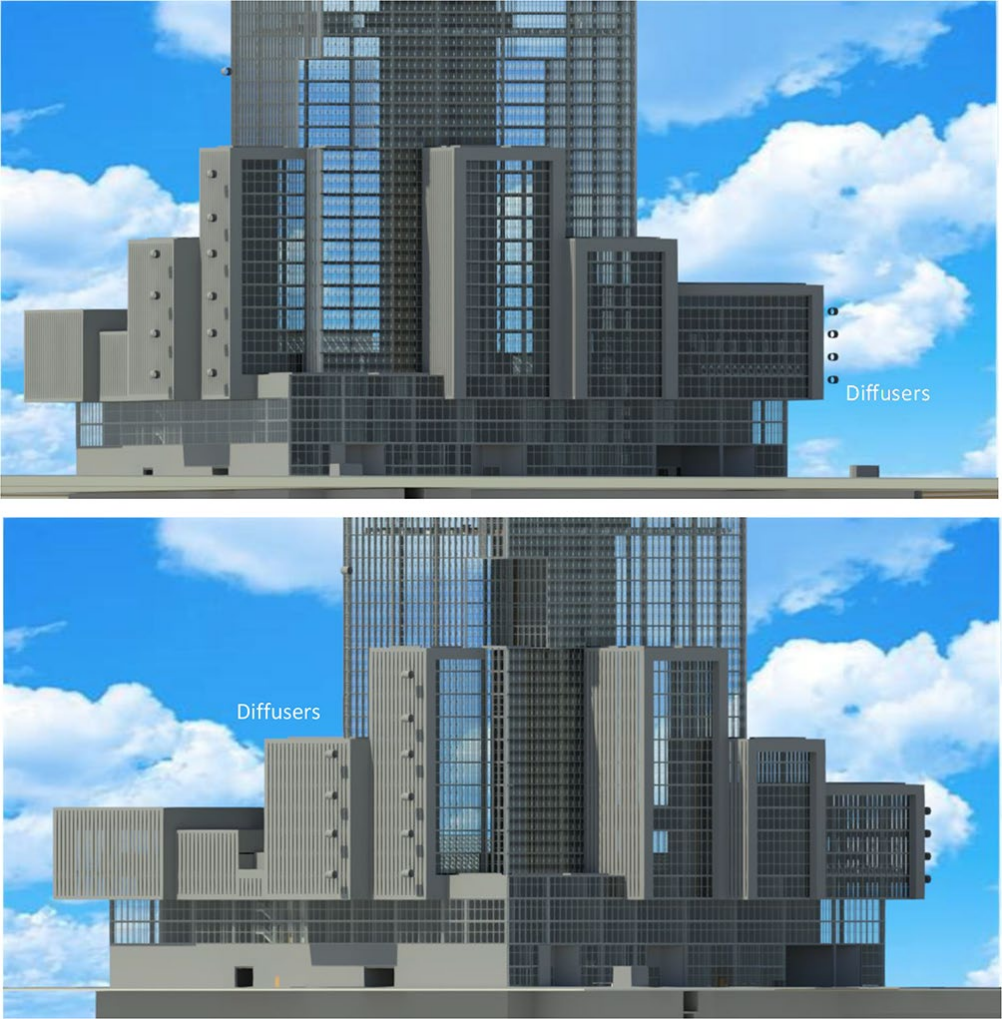
Several optimized diffusers with an area ratio of 1.5 fixed to sides of facades of a tall building development.

When considering diffusers’ building integration prospects we can see that diffusers are one of the least intruding, or building form hampering technologies. Especially as this paper illustrates that the most efficient ones are not necessarily the biggest sized, but those that are reasonably sized through optimization. Figure 9, below shows the optimized diffuser in this study of 1.5 aspect ratio in a proposed tall building design arranged along the height of building’s blocks at a proximity of 90cm to building facades at different levels. Where diffusers will generate accelerated flows that can be used by wind turbines, thus generating more power without being affected by turbulence generated by building blocks. When this happen the building would presumably receive more renewable wind energy. While for performance, due to diffuser’s small size, less vibrations or noise would be generated from wind flows. In addition, diffusers provide barrier for turbine produced noise.

## Conclusion

Diffuser augmented wind flows provide a promising technology to harness wind energy within the built environment. It helps to eliminate the effect of turbulence and variability of wind flows within the Built environment, which is a condition leading to adverse decline in energy output from wind turbines. Despite the fact that all area ratios studied in this paper provided increase in resulting velocities with a range of increase from 120 to 161.25%, flow separation clearly affects the ability for further increase in resulting velocities in line with the findings of Ref.^11^. Unless there is a way to benefit from this separation by special turbine’s blade design for instance.

Power coefficient C_p_ of the majority of studied diffusers ranged from 2 to 3 time the power of a bare turbine under the same incident velocities. While area ratio of 1.5 experienced a power coefficient maximum of 4.2 times that of a bare turbine at incident velocity of 16 m/s. That is because the Area ratio of 1.5 produced the highest resulting velocity of 25.8 m/s under that particular incident velocity. These findings are similar to the results obtained by Refs.^15,20^, and as such, provides a validation to the obtained results. The study concluded that it is possible to obtain considerable wind energy from a relatively small or compact diffuser (i.e., outlet/inlet, 0.75 m/0.50 m) by employing a standard aerofoil cross section. This makes it more favorable for building integration as it will help to reduce the impact of large diffusers on overall buildings’ appearance and performance. As for the appearance, small diffusers easily integrated into the building facades without being obtrusive; while for performance due to its small size less vibrations or noise generated from either wind flows or turbine operation. The study hence filling the gap of to what extent we can increase the area ratio of diffusers to get the maximum power generation and what would be the optimum diffuser size for maximum power generation for building’s integration. A future research query would be towards the prospects of spacing these diffusers across and around building’s envelope and how this would effect on expected power generation.

## Data Availability

All data generated or analysed during this study are included in this published article.
